# Public Health Impact of Reemergence of Rabies, New York

**DOI:** 10.3201/eid0809.010524

**Published:** 2002-09

**Authors:** Hwa-Gan H. Chang, Millicent Eidson, Candace Noonan-Toly, Charles V. Trimarchi, Robert Rudd, Barbara J. Wallace, Perry F. Smith, Dale L. Morse

**Affiliations:** * New York State Department of Health, Albany, New York, USA; † School of Public Health, University at Albany, Albany, New York, USA

**Keywords:** rabies, epidemiology, vaccination

## Abstract

This report summarizes the spread of a raccoon rabies epizootic into New York in the 1990s, the species of animals affected, and human postexposure treatments (PET). A total of 57,008 specimens were submitted to the state laboratory from 1993 to 1998; 8,858 (16%) animals were confirmed rabid, with raccoons the most common species (75%). After exposure to 11,769 animals, 18,238 (45%) persons received PET, mostly because of contact with saliva or nervous tissue. We analyzed expenditure reports to estimate the cost of rabies prevention activities. An estimated $13.9 million was spent in New York State to prevent rabies from 1993 to 1998. Traditional prevention methods such as vaccinating pets, avoiding wildlife, and verifying an animal’s rabies status must be continued to reduce costly PET. To reduce rabid animals, exposures, and costs, oral vaccination of wildlife should also be considered.

The incidence of human rabies is high in developing countries, and most cases of the illness occur in humans with untreated dog bites (1,2). In developing countries, rabies control in domestic canids has shifted the source of rabies exposures for most humans and domestic animals to wild terrestrial animals. Reported animal rabies cases in the United States have increased dramatically since 1990 in association with the raccoon rabies epizootic in the mid-Atlantic and northeastern states. Before 1990, rabies infections in New York were attributed to red fox and bat variants of the virus. After 1993, rabies testing indicated that the red fox variant no longer existed in the state (3); instead, a raccoon rabies variant had moved into New York State from Pennsylvania in 1990.

Nationwide, the number of reported rabies cases in animals increased from 6,972 in 1991 to 9,495 in 1993, but decreased to 8,224 in 1994, 8,509 in 1997, and 7,961 in 1998 (4–11). Wild animals accounted for 92% of animal rabies cases in the United States, with raccoons reported most frequently, followed by skunks, bats, and foxes. The number of human cases remained low in the same time period, ranging from one case in 1998 to six cases in 1994 (4–11). In 1991, New York State accounted for 14% of reported rabid animals in the United States; this proportion increased to 28% in 1993 (12,13).

 The exposure of humans and domestic animals to rabid animals has resulted in an estimated 16,000–39,000 persons per year receiving postexposure prophylaxis treatment (PET) in the United States (14). The estimated cost for human postexposure treatment ranges from $1,039 to $4,447 per person (15). Including pet animal vaccinations, the total cost of treatment was recently estimated at $300 million per year in the United States (16).

New York State has passed a legislative appropriation for rabies prevention and PET. Reimbursement of PET costs not covered by third-party payers was first established more than 50 years ago in response to concerns about potential human deaths from fox rabies in those who could not afford treatment. Since the New York State Department of Health (NYSDOH) disburses these funds, this agency can provide accurate estimates of the cost of postexposure rabies treatments in the state. In addition, NYSDOH’s active rabies laboratory conducts all diagnostic work in the state, excluding New York City, which has its own laboratory (although test result data from New York City are also reported).

Initial analyses of rabies treatments for four New York counties in 1993 and 1994 have been previously published (13). In this study, we examine the reemergence of rabies in New York and summarize information on the spread of rabid animals, the type of animals involved, trends in human exposures to rabid animals, and the intervention strategies to reduce human exposures from 1993 to 1998.

## Materials and Methods

In New York State, public health law requires health-care providers with knowledge of a person exposed to an animal suspected of having rabies infection to report the incident to the local health unit (LHU). LHUs are required to have comprehensive rabies control protocols that provide 24-hour availability of county staff to manage possible exposures, including 10-day confinement and observation of apparently healthy dogs and cats responsible for exposures; collection, preparation, and submission of animal specimens to the rabies laboratory for prompt rabies examination; authorization of human PET; and provision of pet vaccination clinics. Annually, LHUs must submit to NYSDOH a detailed expenditure report for state-reimbursed costs including PET, laboratory specimen preparation, and pet vaccination clinics. We used fiscal year data (April–March) from 1993 to 1998 to estimate the overall cost of human PETs in New York.

A rabies specimen history form accompanies each animal specimen submitted to the New York State Wadsworth Center rabies laboratory for testing. Using this form, we gathered information specific to the specimen regarding species, location of capture, nature of human and animal contacts, and rabies testing results.

 A rabies surveillance report form is completed by the LHU for each animal exposure that resulted in human postexposure treatment and for each rabid animal. These surveillance forms are forwarded to the NYSDOH Bureau of Communicable Disease Control for data entry and analysis. Data collected on these reports include animal species, location, type of exposure, and number of humans exposed to the suspected animal.

We matched data from the surveillance reports with data from rabies laboratory specimen history reports. Positive test results with missing surveillance information were actively followed up with LHUs to assure the completeness of exposure and treatment data. The data from laboratory and human exposure reports have been computerized for the years 1993–1998 and are analyzed in this report. To map New York’s counties and the year raccoon rabies was first confirmed in each county, we included data from 1991 to 1997.

## Results

**Figure 1:**
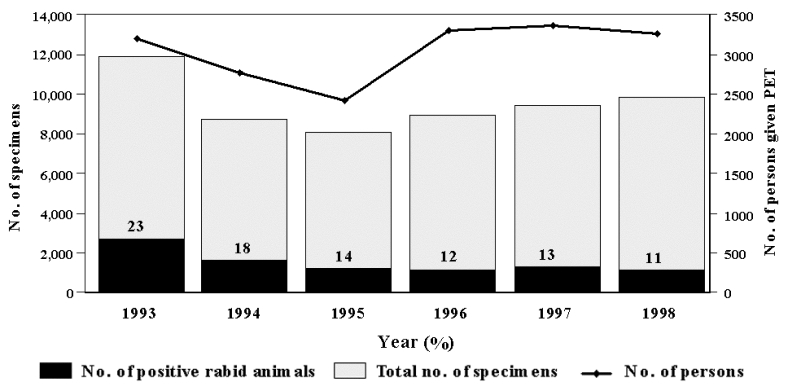
Number of animal specimens tested for rabies, rabid animals, and humans receiving postexposure treatments, New York, 1993–1998.

 From 1993 to 1998, a total of 56,947 animal and 61 human specimens were submitted for rabies testing, with the highest number of tested animals in 1993 (11,896) and the lowest in 1995 (8,032) (Figure 1). The overall proportion of tested animal specimens with laboratory-confirmed rabies virus was 16%, and the number of rabid animals declined from a high of 2,688 (23%) in 1993 to 1,097 (11%) in 1998. However, we did not observe a similar decline in the 18,238 humans who received PET during this period, with the highest number in 1997 (3,373) and the lowest in 1995 (2,422).

**Figure 2:**
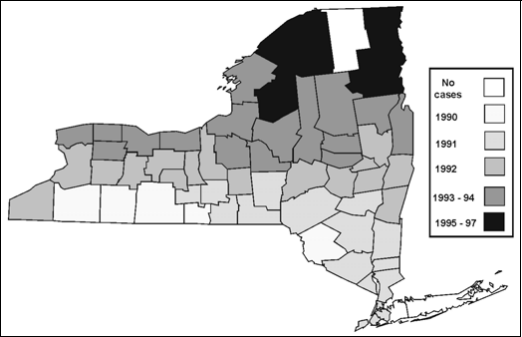
Annual distribution of raccoon-variant rabies when first confirmed within each county, New York, 1991–1997.

 The geographic movement of raccoon variant of rabies is shown in Figure 2, which indicates when the variant was first confirmed in each county from 1991 to 1997. By 1998, only three counties reported no rabid raccoons or other animals infected with the raccoon variant of rabies. Although the raccoon rabies variant continued to spread throughout the state in the 1990s, the annual number of raccoons testing positive for rabies decreased from 2,318 in 1993 to 691 in 1998.

**Figure 3:**
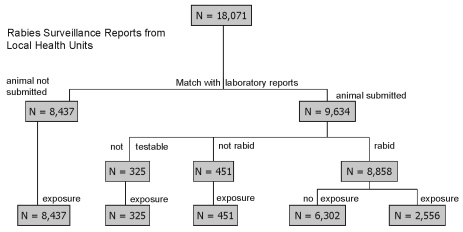
Matching of rabies surveillance reports from local health departments and laboratory reports for submitted animals by test result and human exposure, New York, 1993–1998.

 From 1993 to 1998, a total of 18,071 animal rabies surveillance reports were received from local health departments (Figure 3). Of these, 8,437 (47%) were for exposures to animals not submitted for rabies testing. The annual number of surveillance reports without an animal submitted for rabies testing increased from 1,194 in 1993 to 1,714 in 1998.

A total of 8,858 rabies surveillance reports were received on animal specimens with laboratory-confirmed rabies (Figure 3), with 6,302 representing rabid animals in which no human exposure was reported. Of the number of rabid animals associated with human exposures, the species most frequently involved were raccoons (65.2%), skunks (10.4%), bats (7.2%), cats (6.5%), and foxes (5%) (Table 1). For some domestic species, a large proportion of the rabid animals were reported to have exposures resulting in human PET, such as goats (100%), horses (96%), cats (90%), dogs (87%), and cows (82%). High numbers of PETs (>10) for single incidents of rabid animals were documented for many animal species, including raccoons, bats, foxes, cats, cows, deer, dogs, horses, and ferrets. In 1996, 465 persons who attended a county fair received PET because of contact with one rabid goat.

**Table 1:** Rabid animals by species, human exposure, and postexposure treatment, New York, 1993–1998^a^

Animal species	No. (%)of rabid animals		No. (range)^b^ of humans receiving PET
	without human exposure	with human exposure	
Raccoon Skunk Bat Fox Cat Cow Woodchuck Deer Dog Horse Beaver Goat Bobcat Coyote Rabbit Sheep Ferret Other^c^ Total	4,983 (79.1) 895 (14.2) 221 (3.5) 101 (1.6) 18 (0.3) 12 (0.8) 44 (0.7) 14 (0.2) 3 (<0.1) 1 (<0.1) 2 (<0.1) 0 0 1 (<0.1) 1 (<0.1) 0 0 6 (<0.1) 6,302 (100)	1,666 (65.2) 266 (10.4) 184 (7.2) 127 (5.0) 166 (6.5) 54 (2.1) 21 (0.8) 10 (0.4) 20 (0.8) 22 (0.9) 4 (0.2) 4 (0.2) 3 (<0.1) 2 (<0.1) 2 (<0.1) 2 (<0.1) 2 (<0.1) 1 (<0.1) 2,556 (100)	2,944 (1–25) 470 (1–8) 377 (1–12) 229 (1–10) 844 (1–36) 246 (1–30) 32 (1–5) 42 (1–13) 286 (1–37) 139 (1–14) 9 (1–3) 476 (1–465) 7 (1–4) 2 (1) 12 (5–7) 7 (2–5) 16 (3–13) 1 (1) 6,139 (1–465)

^a^PET, postexposure treatment. ^b^Range of number of PETs for a single exposure incident for a rabid animal ^c^Other species included one rabid opossum resulting in human PET, three rabid opossums, one fisher, one pig, and one otter without consequent human PET.

A total of 11,552 persons received PET for exposure to 8,762 animals with specimens unavailable for testing or not testable because of specimen condition (Table 2). In addition, 547 persons received PET for exposure to 451 animals that had negative rabies virus tests (Table 2). Cats, bats, and dogs each accounted for approximately 25% of the exposures requiring treatment when the suspect animal was unavailable for testing, with raccoons accounting for more than 10% of the exposures. Similarly, these species also accounted for most of the PETs when laboratory testing confirmed that the animal was not rabid, although more than 40% of the unnecessary treatments resulted from cat exposures.

**Table 2:** Nonrabid or suspected rabid animals and the number of humans receiving postexposure treatment, by animal species, New York, 1993–1998^a^

Animal species	No. (%) of suspected rabid animals^b^	No. (range^c^) of humans receiving PET	No. (%) of nonrabid animals	No. (range^c^) of humans receiving PET
Cat Bat Dog Raccoon Skunk Fox Woodchuck Squirrel Opossum Deer Muskrat Cow Rabbit Rat Ferret Chipmunk Other Unknown Total	2,373 (27.1) 2,289 (26.1) 2,000 (22.8) 952 (10.9) 160 (1.8) 104 (1.2) 92 (1.1) 73 (0.8) 48 (0.5) 26 (0.3) 15 (0.2) 18 (0.2) 17 (0.2) 16 (0.2) 12 (0.1) 12 (0.2) 104 (1.2) 450 (5.1) 8762 (100)	2,620 (1–11) 4,181 (1–40) 2,067 (1–15) 1,247 (1– 21) 211 (1–6) 125 (1–3) 99 (1–3) 75 (1–2) 51 (1–2) 32 (1–4) 15 (1 ) 37 (1–2) 18 (1–2) 16 (1) 16 (1–3) 13 (1–2) 133 (1–7) 596 (1–7) 11552 (1–40)	183 (40.6) 116 (25.7) 49 (10.9) 51 (11.3) 10 (2.2) 6 (1.3) 9 (2.0) 6 (1.3) 2 (0.4) 3 (0.7) 5 (1.1) 1 (0.2) 1 (0.2) 2 (0.4) 3 (0.7) 1 (0.2) 3 (1.2) 0 451 (100)	220 (1–5) 148 (1–4) 53 (1–3) 67 (1–5) 12 (1–3) 6 (1) 9 (1) 6 (1) 1 (1) 5 (1–2) 6 (1–2) 1 (1) 1 (1) 2 (1) 3 (1) 1 (1) 5 (1) 0 547 (1–5)

^a^PET; postexposure treatment. ^b^Rabies status of animals could not be determined by testing (animal not submitted for rabies testing or specimen not testable because of specimen condition) ^c^Range of number of PETs for a single exposure incident to a potentially rabid animal.

Across all categories of rabies status for the animal, most postexposure treatments were provided because of possible contact with saliva or nervous tissue (44.5%), followed by bite (34.9%) and scratch (5.8%) exposures (Table 3). When the animal tested positive for rabies, a larger proportion of the PETs (82.9%) were for saliva or nervous tissue contact, particularly from raccoons. In contrast, for the suspect or nonrabid animals, most of the PETs were for bite exposures (47.6% and 60%, respectively).

**Table 3:** Number of humans receiving postexposure treatment, by animal status and type of exposure, New York, 1993–1998

Type of exposure	No. (%) of humans receiving PET ^a^			
	Rabid animal	Suspect rabid animal^b^	Nonrabid animal^c^	Total
Bite	538 (.8)	5,503 (47.6)	328 (60.0)	6,369 (34.9)
Scratch	224 (3.6)	773 (6.7)	56 (10.2)	1,053 (5.8)
Contact with saliva	5,090 (82.9)	2,891 (25.0)	131 (23.9)	8,112 (44.5)
Unknown exposure	287 (4.8)	2,385 (20.6)	32 (5.9)	2,704 (14.8)
Total	6,139 (10)	11,552 (100)	547 (100)	18,238 (100)

^a^PET, postexposure treatment. ^b^PETs due to exposure to animals not submitted for rabies testing or specimen was not testable because of specimen condition. ^c^PETs due to exposure to animals that tested negative for rabies.

 Two fatal human rabies cases related to bat exposure occurred in New York in 1993 and 1995 (the 1995 case was in a Connecticut resident hospitalized in New York) resulting in treatment of 55 and 48 persons, respectively, who had contact with the cases either at home or in the hospital. Although bats represented only 4.6% of the rabid animals in New York, exposure to bats accounted for 25.8% of the PETs, with a total of 4,706 persons receiving PET after exposure to bats in the state. Fifty-one percent of the bat-related PETs were classified as “unknown” in regard to exposure, and 28% were provided because of reported contact with saliva or nervous tissue.

 The total expenditure for PETs, laboratory specimen preparation, and pet vaccination clinics increased in New York from $1.8 million in the 1993–1994 fiscal year to $2.9 million in the 1998–1999 fiscal year (Table 4). The estimated average annual statewide cost for the biologics and administration of the PETs was $1.8 million; the average cost per person for PET was $927, increasing from $769 in the 1993 fiscal year to $1,136 in the 1998 fiscal year.

**Table 4:** Rabies expenditures for postexposure treatments, laboratory specimen preparation, and pet vaccination clinics, by fiscal year,^a^ New York, 1993–1998

Type of expenditure	1993–1994	1994–1995	1995–1996	1996–1997	1997–1998	1998–1999
PET^b^	$1,222,125	$1,919,606	$1,257,621	$1,835,058	$2,092,572	$2,347,555
State	$669,564	$1,006,471	$679,902	$311,356	$974,079	$959,362
Local	$138,415	$170,284	$116,368	$787,500	$84,630	$188,723
Other	$414,146	$742,851	$461,351	$736,202	$1,033,863	$1,199,470
Average per person^c^	$769	$822	$824	$944	$1,020	$1,136
Specimens^d^	$265,037	$256,518	$251,796	$246,794	$276,219	$270,184
State	$200,702	$234,097	$231,917	$226,224	$254,888	$250,762
Local	$64,335	$22,421	$19,879	$20,570	$21,331	$19,422
Clinics^e^	$271,062	$328,532	$294,251	$289,729	$244,254	$262,351
State	$84,671	$167,763	$139,456	$117,840	$110,145	$118,002
Local	$186,391	$160,769	$154,795	$171,889	$134,109	$144,349

^a^Fiscal year is April – March. ^b^PET, postexposure treatment; PET costs incurred by the New York State Department of Health (<$1,000 per PET), the local health departments, and others (third-party payers). ^c^Calculated by dividing total treatment costs by number of persons treated. ^d^Laboratory specimen preparation costs incurred by the New York State Department of Health (limits of $60 per small animal specimen and $75 per livestock specimen) and the local health departments, for animal euthanasia, head removal, and specimen shipment. ^e^Pet vaccination clinic costs incurred by the New York State Department of Health (limits of five clinics per year and $1,000 per clinic) and local health departments.

## Discussion

 The public health impact of the reemergence of rabies in New York resulting from the spread of raccoon variant in the 1990s was profound in terms of the number of rabid animals diagnosed, humans exposed and treated, and PET costs. Despite the decreasing number of rabid animals during the study period, the increasing number of humans receiving treatment for rabies from 1993 to 1998 appeared to be a result of the high number of suspected rabid animals (untested) and the high number of reported bat exposures following publicity surrounding two bat rabies–related human deaths.

 The high proportion of PETs associated with exposures other than bites (9,165/18,238 [50%]) in our review indicates the degree of human fear about possible rabies and the difficulties in interpreting definitions of exposure (17,18). This concern is also indicated by the PET administered to 465 persons exposed to a rabid goat in 1996 and 547 persons exposed to animals that tested negative for rabies from 1993 to 1998. With 41% of 11,552 treated persons exposed to dogs and cats without testable specimens (because of specimen condition), efforts to find these pets to verify their rabies status may be helpful in reducing unnecessary treatments (19). The annual reviews and recommendations on animal rabies control from the National Association of State Public Health Veterinarians (20) should be applied to reduce human exposures to rabid animals and unnecessary rabies PETs.

 A few studies suggest that >$1 billion per year has been spent recently to prevent rabies in the United States (14), with the vaccination of pet animals accounting for 82% of the expenditures (the cost associated with pet vaccination given by private providers was not available for our study). Our study estimated that $13.9 million was spent to prevent rabies in New York, where $10.7 million (77%) was used on PET from 1993 to 1998. The use of PET for 547 persons exposed to nonrabid animals supports the need for better education of health-care providers to determine whether PET is really necessary pending laboratory test results and the need for public education to reduce exposure to rabid animals and minimize contact to exposed pets.

 Seventy-five percent (24/32) of the human rabies cases in the United States since 1990 have been attributed to bat variants (21–23). The two bat-variant deaths in New York exemplify the new realization that human rabies may result from encounters with bats when bites are unreported or unrecognized. In July 1993, a child without a history of a known bite or other exposure to a suspected rabid animal died from rabies that was identified as a bat variant (24). In October 1995, a Connecticut resident without history of animal bites but possible exposure to a bat died from rabies in a N. Y. hospital and resulted in 48 state residents receiving PET (25). Bat exposures accounted for 25% of New York’s PETs from 1993 to 1998, underscoring the importance of avoiding contact with bats and the need to test bats for rabies when human contact may have occurred (26).

 The persistence and spread of rabies in raccoons and domestic animal exposure to this variant continue to be an important issue for public health officials. The reemergence of wildlife rabies in areas like New York (after the fox variant had moved out of the state) as a result of the unimpeded northward spread of the raccoon variant into the state and increased recognition of the importance of bat variants has led to a large number of rabies cases both in domestic and wildlife species and a corresponding number of human rabies PETs. Traditional public health methods of surveillance, public and provider education to avoid exposure to potentially rabid animals, appropriate postexposure prophylaxis, and emphasis on verifying the negative rabies status of suspect animals to avoid unnecessary treatments will remain important methods for rabies control. However, the major impact of raccoon rabies in human exposure and treatments may also need to be addressed with new wildlife rabies control methods such as oral rabies vaccine (27–29).
